# Cellular Localization of *gdnf* in Adult Zebrafish Brain

**DOI:** 10.3390/brainsci10050286

**Published:** 2020-05-11

**Authors:** Chee Ern David Wong, Khang Hua, Simon Monis, Anwar Norazit, Suzita Mohd Noor, Marc Ekker

**Affiliations:** 1 Department of Biomedical Science, Faculty of Medicine, University of Malaya, Kuala Lumpur 50603, Malaysia; davidwce89@gmail.com (C.E.D.W.); anwar.norazit@um.edu.my (A.N.); suzita@um.edu.my (S.M.N.); 2 Department of Biology, Faculty of Science, University of Ottawa, Ottawa, ON K1N 6N5, Canada; Khanghua88@gmail.com (K.H.); smoni067@uottawa.ca (S.M.)

**Keywords:** *gdnf*, radial glial cells, Sox2, neuronal progenitor cells, *dlx*, GABAergic neurons, dopaminergic neurons

## Abstract

Glial cell line-derived neurotrophic factor (GDNF) was initially described as important for dopaminergic neuronal survival and is involved in many other essential functions in the central nervous system. Characterization of GDNF phenotype in mammals is well described; however, studies in non-mammalian vertebrate models are scarce. Here, we characterized the anatomical distribution of *gdnf*-expressing cells in adult zebrafish brain by means of combined in situ hybridization (ISH) and immunohistochemistry. Our results revealed that *gdnf* was widely dispersed in the brain. *gdnf* transcripts were co-localized with radial glial cells along the ventricular area of the telencephalon and in the hypothalamus. Interestingly, Sox2 positive cells expressed *gdnf* in the neuronal layer but not in the ventricular zone of the telencephalon. A subset of GABAergic precursor cells labeled with *dlx6a-1.4kbdlx5a/6a:* green fluorescence protein (GFP) in the pallium, parvocellular preoptic nucleus, and the anterior and dorsal zones of the periventricular hypothalamus also showed expression with *gdnf* mRNA. In addition, *gdnf* signals were detected in subsets of dopaminergic neurons, including those in the ventral diencephalon, similar to what is seen in mammalian brain. Our work extends our knowledge of *gdnf* action sites and suggests a potential role for *gdnf* in adult brain neurogenesis and regeneration.

## 1. Introduction

Glial cell line-derived neurotrophic factor (GDNF) is the most studied member of the GDNF family of ligands (GFLs), which also consists of neurturin, artemin, and persephin. GDNF has a high affinity toward the GDNF family receptors (GFR)α1, which then activates the intracellular signaling cascades such as MAP kinases and Akt through the receptor tyrosine kinase RET and/or the neural cell adhesion molecule [1,2,3]. GDNF was first identified from conditioned media of striatal astrocytes, and *in vitro* studies showed GDNF could prevent apoptosis and enhance differentiation of embryonic mesencephalic-derived dopaminergic neurons [4]. Extensive preclinical research carried out on GDNF for its restorative function in Parkinson’s Disease (PD) [5,6,7,8] and its crucial role for the maintenance of adult catecholaminergic neurons in the nigrostriatal system [9] have shown promise and generated great interest in using GDNF as a therapeutic agent for intervention in neurodegenerative diseases such as PD.

GDNF is widely expressed in the central and peripheral nervous systems such as motor neurons [10] as well as in the enteric nervous system [11]. The presence of GDNF in these neurons is correlated with its significant role in neuroprotection. Study of tissue-specific GDNF expression in the developing human fetal brain suggests critical importance in the development and maintenance of various types of neuronal and non-neuronal cells [12]. During mouse development, *gdnf* transcripts first appear in the ventral forebrain at E7.5, with expression peaking at E.9.5, then decreasing from E10.5. At E.13.5, *gdnf* expression increases but only in the ventral midbrain. Interestingly, *gdnf* expression re-emerges throughout the brain at 18.5 and persists into adulthood [13,14]. 

Due to the sheer complexity of the mammalian brain, there is still no consensus on the endogenous functions of GDNF on dopaminergic neuron development and maintenance [9,15,16,17,18], and a recent clinical study showed contrasting results [19]. The zebrafish (*Danio rerio*) has been recognized as an alternative model for studying molecular and cell biology in neuroscience and translational research [20]. Given that the zebrafish has simpler neuroanatomy and smaller brain size without compromising its homology of neural circuit function, this model may contribute some clarity of the mode of action of *gdnf* on the neurophysiology of the brain [21]. For instance, fewer neurons in the zebrafish brain allow for qualitative analyses of neuronal activity patterns in order to reconstruct the dynamic brain network into neuronal computation information to decipher mechanistic insights underlying higher-level vertebrate brain functions. These properties contribute to the use of the zebrafish in examining the reparative capability of the brain. 

Shepherd et al. were the first to characterize *gdnf* in zebrafish. Whole-mount in situ hybridization showed that *gdnf* was expressed in the central nervous system (CNS) of zebrafish larvae as well as in the enteric neurons and pronephric ducts [22]. As reported for rodent *Gdnf*, zebrafish *gdnf* was shown to be critical in enteric nervous system development and peripheral axons of sensory neurons [22,23]. Moreover, neuroprotection against neuronal death induced by a mutated human Tau protein was seen in zebrafish overexpressing *gdnf* [24]. Nevertheless, limited *gdnf* functional studies have been carried out in the zebrafish central nervous system, particularly in the brain itself at both larval and adult stages. Expression of *gdnf* and its receptor has been documented in the adult zebrafish brain, however, the types of cells expressing *gdnf* have not yet been reported. This information is essential to add further knowledge on the potential functions or underlying mechanisms of *gdnf* action in the CNS of zebrafish. Here, we characterize the neuroanatomical expression of *gdnf* and identify the *gdnf*-positive cells in the adult zebrafish brain. 

## 2. Materials and Methods

### 2.1. Animal Care and Handling

All zebrafish used in this study were housed under standard conditions with 14-hours light and 10-hours dark of photoperiod, and water temperature set at 28 °C. The experiment handling and euthanasia procedures were approved by the University of Ottawa Animal Care Committee following guidelines of the Canadian Council on Animal Care, under the ethics number: BL-2081. Zebrafish did not receive any drug treatment prior to the experiment. Wild-type and transgenic Tg (*dlx6a-1.4kbdlx5a/6a:*GFP) [25] were used in this study. 

### 2.2. Brain Dissection and Tissue Processing

Sexually mature adult zebrafish were euthanized with an overdose of tricaine methanesulfonate (Sigma-Aldrich). Brains from at least two adult zebrafish were used for the analysis of each neuronal protein maker in this study. The skull was then opened to expose the brain and fixed overnight in 4% paraformaldehyde (PFA) at 4 °C. The brain was dissected and washed with phosphate-buffered saline (PBS) followed by overnight cryopreservation in 30% sucrose at 4 °C. The brain was embedded in optimal cutting temperature (OCT) compound and flash frozen in liquid nitrogen. The tissue was cryosectioned into ~12–14 µm thick sections that were adhered to the Superfrost Plus^TM^ coated slides (Thermo Scientific). The slides were stored at −20 °C until further use.

### 2.3. In Situ Hybridization 

RNA template was isolated from 7 dpf larvae for *gdnf* probe synthesis. Reverse transcriptase-polymerase chain reaction (RT-PCR) amplification of the targeted *gdnf* coding region used the following primers: 5’-TGTCCACACGTCCCCTTTTC–3’ (forward) and reverse primer 5’–CTCCAAGCTGTCGTCCAGAA–3’ (reverse). The PCR products were TA-cloned into pDRIVE vector (Qiagen) and the sequencing analysis was carried out to confirm the product sequence. The plasmid was linearized by *Hin*dIII or *Bam*HI and digoxigenin (DIG)-labeled *gdnf* sense and antisense probes were generated, respectively, by *in vitro* transcription using DIG RNA labeling mix (Roche) contained T7 or SP6 RNA polymerase (Roche). 

In situ hybridization was performed according to Reference [26] with slight modifications. To minimize the possibility of RNA degradation, all buffers used before the probe hybridization step were prepared in diethylpyrocarbonate (DPEC)-treated water or PBS. Briefly, the sections were washed with 0.3% Triton-X and PBS. The tissues were permeabilized with proteinase K (5 µg/mL) (0.1 M Tris-HCl PH 8, 50 mM EDTA) for 15 min at room temperature. After re-fixation in 4% PFA, the tissues underwent an acetylation step to reduce the background. The tissues were then incubated with hybridization buffer (50% deionized formamide, 10% dextran sulfate, 1 mg/mL yeast tRNA, 1X Denhardt’s, 1X salt) containing DIG-labeled *gdnf* probe overnight at 70 °C in a humidified chamber. On the following day, the slides were washed in solution A (1X SSC, 50% formamide, 0.1% Triton-X) at 70 °C for twice, and twice again with TBST (0.14 M NaCl, 2.7 mM KCl, 25 mM Tris HCl PH 7.5, 0.1% Triton-X) at room temperature. The tissues were incubated in the blocking solution (10% FBS/TBST) for 1 hour at room temperature. The anti-digoxigenin alkaline phosphatase Fab fragments (1:2000, Roche) were diluted in blocking solution and incubated with the sections overnight at 4 °C. On the next day, the tissues were washed with TBST and NTMT (100 mM NaCl, 100 mM Tris-HCl, 50 mM MgCl_2_, 0.1% Triton-X) before it stained with substrate solution (NBT/BCIP, Roche) to reveal the hybridization signal. The signal was closely monitored to avoid overstaining. The tissues were stained overnight at room temperature if the staining was still weak after 3–4 h incubation in substrate solution. 

### 2.4. Combined Fluorescence in Situ Hybridization and Immunohistochemistry 

The protocol for fluorescence in situ hybridization (FISH) was slightly modified from the colorimetric in situ hybridization (ISH) procedure. The tissue permeabilization step using proteinase K was replaced with incubation in 10 mM sodium citrate (0.05% Tween-20) for 20 min at 85 °C and the hybridization temperature was adjusted to 63 °C. Endogenous horseradish peroxidase was inhibited by incubating in 2% H_2_O_2_/TNT (0.1 M Tris-HCl pH 7.5, 0.15 M NaCl, 0.5% Triton-X) for 10 min. The sections were then incubated overnight with anti-DIG-POD (ROCHE) in blocking solution (0.1 M Tris-HCl pH 7.5, 0.15 M NaCl, 0.5% Tween-20, 0.5% Perkin Elmer block powder) at room temperature. On the following day, the sections were washed several times in TNT buffer and the fluorescent in situ signal was amplified using the Tyramide Signal Amplification (TSA) Cyanine 3 system (PerkinElmer, NEL753001KT) according to the manufacturer’s instructions. Cy3 fluorophore was diluted (1:100) in amplification solution and the tissues were stained for 20 min in the dark. 

Immunohistochemistry was carried out immediately after FISH. To stop the FISH reaction, sections were washed in PBS and re-fixed for 5 min in 4% PFA/PBS. After rinsing with 0.5% PBST (PBS/0.5% Triton-X), the tissues were treated with 5% FBS/PBST for 1–2 h at room temperature and incubated overnight at room temperature with different primary antibodies (diluted in 5% FBS/PBST) as listed in Table 1. Sections were washed in PBST and incubated overnight at 4 °C with either goat anti-rabbit or anti-mouse Alexa Fluor 488 (1:400, Invitrogen). After secondary antibody incubation, the sections were washed with PBST and then counterstained with Vectashield mounting medium with DAPI (Vector Laboratories). A sense probe was used as a specificity control.

### 2.5. Microscopy 

At least 5 sections per region with *gdnf* signal were examined and the most representative images were captured. There was no obvious difference in the staining patterns between the samples. All sections were imaged under the Nikon A1 confocal microscope with 25× water-dipping objective and with digital zoom for close-up analysis. Z-stacks of 1–2 µm were captured and the low magnification images were constructed from the maximum intensity projection of z-stacks of the section using NIS-Elements software (version 4.5, Praha, Czech Republic. Higher magnification images were used for co-localization analysis; they were constructed from selected z-stacks. Co-localization analysis was carried out in a qualitative format. The images were post-processed to adjust for light and contrast using the NIS-Elements software (version 4.5) or Image J. All nomenclature and graphical illustrations images of coronal and sagittal sections of the adult zebrafish brain are adapted from “Neuroanatomy of The Zebrafish Brain” [27] unless stated otherwise.

## 3. Results

### 3.1. Characterization of gdnf Expression Patterns in the Adult Zebrafish Brain

**Figure 1:**
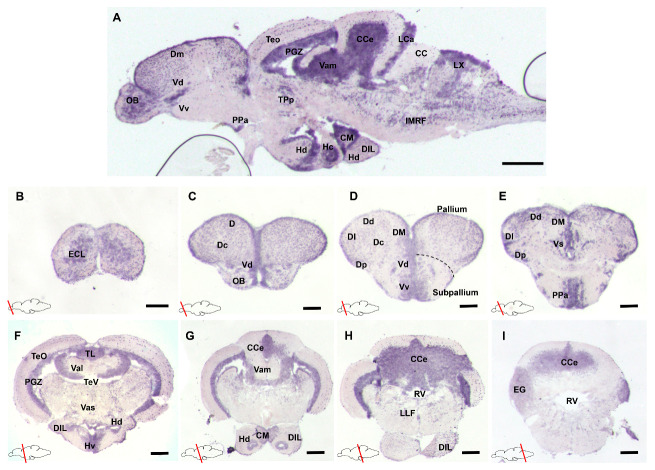
Glial cell line-derived neurotrophic factor *(gdnf)* expression pattern in the adult zebrafish brain (**A**) Sagittal section of the adult zebrafish brain showing distribution of *gdnf* mRNA by in situ hybridization in, (**B**–**E**) telencephalon, (**F**–**H**) mesencephalon, and (**I**) rhombencephalon. Scale bars: (**A**) 200 µm (**B**–**I**) 50 µm. Abbreviations: CC: Crista cerebellaris; CCe: Corpus cerebelli; CM: Corpus mamillare; D: Dorsal telencephalic area; DI: later zone of dorsal telencephalic area; DIL: Diffuse nucleus of the inferior lobe; Dm: Medial zone of dorsal telencephalic area; Dc: Central zone of dorsal telencephalic area; Dd: Dorsal zone of dorsal telencephalic area; Dp: Posterior zone of dorsal telencephalic area; ECL: External cellular layer of olfactory bulb including mitral cells; EG: Eminentia granularis; Hc: Caudal zone of periventricular hypothalamus; Hd: Dorsal zone of periventricular hypothalamus; IMRF: Intermediate reticular formation; LCa: Lobus caudalis cerebelli DIL; LLF: Lateral longitudinal fascicle; LX: Vagal love; OB: Olfactory bulb; PGZ: Periventricular gray zone of optic tectum; PPa: Parvocellular preoptic nucleus, anterior part; RV: Rhombencephalic ventricle; TeO: Tectum opticum; TeV: Tectal ventricle; TL: Torus longitudinalis; TPp: Periventricular nucleus of posterior tuberculum; Val: Lateral division of valvular cerebelli; Vam: Medial division of valvular cerebelli; Vas: Vascular lacuna of area postrema; Vd: Dorsal nucleus of ventral telencephalic area; VM: ventromedial thalami nuclei; Vs: Supracommissural nucleus of ventral telencephalic area; Vv: Ventral nucleus of ventral telencephalic area.

To analyze the spatial distribution of *gdnf* mRNA, in situ hybridization (ISH) was performed on a series of sagittal or transverse sections of the whole adult zebrafish brain. The results revealed that *gdnf* expression is widely distributed in the adult brain (Figure 1A). In the forebrain, strong *gdnf* signals were detected in the olfactory bulb (OB) (Figure 1B), dorsal (Dd and Dm), and ventral (Vd and Vv) telencephalic area (Figure 1C–E). In the midbrain, there was relatively weaker *gdnf* expression in the optic tectum (TeO) and more intense expression in the periventricular gray zone (PGZ), torus longitudinalis (TL), and periventricular hypothalamus (Figure 1A, F–G). The cerebellum (CCe) in the hindbrain also showed *gdnf* expression (Figure 1H–I).

### 3.2. Characterization of gdnf Expression in Stem/Progenitor Cells

**Figure 2:**
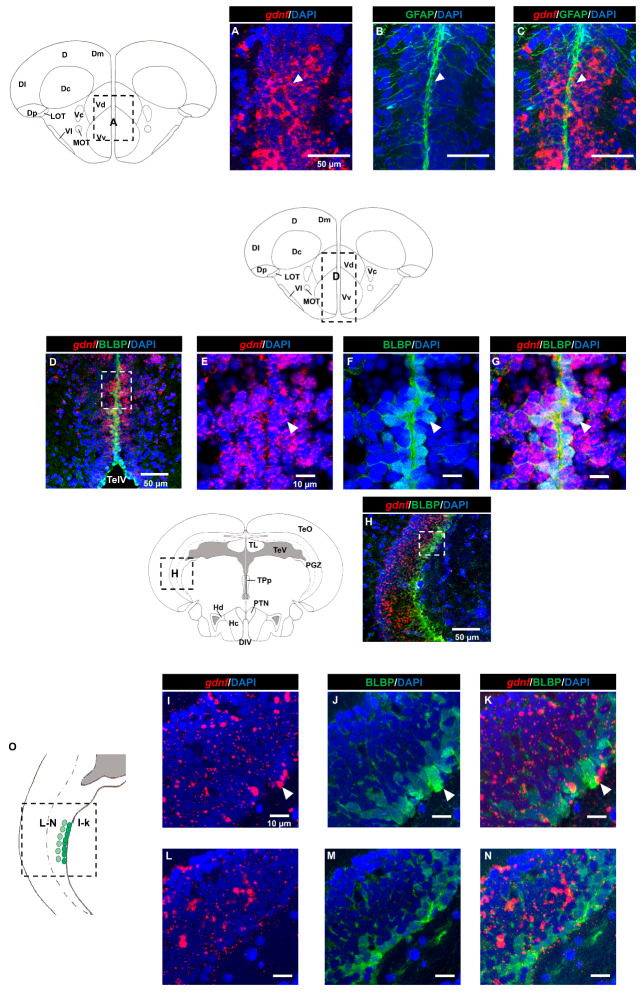

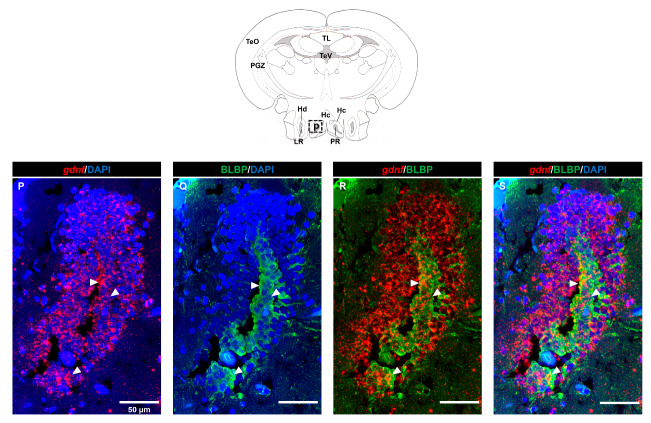
Co-expression of *gdnf* and radial glial cell markers using FISH/immunohistochemistry. (**A**–**C**) *gdnf* is expressed with glial fibrillary acidic protein (GFAP) and (**D**–**G**) brain lipid-binding protein (BLBP) along the ventral telencephalic area, as indicated by the white arrowheads, (**H**) but only few cells with BLBP in the lateral margin of the periventricular gray zone (PGZ) of the optic tectum in the mesencephalon. Enlarged regions of PGZ with different layers of selected z-stacks that consist of BLBP-positive cells in the (**I**–**K**) outer layer and (**L**–**N**) inner layer and that are depicted in the (**O**) schematic diagram. (**P**–**S**) *gdnf* mRNA-positive cells are also BLBP immunoreactive in the caudal zone of periventricular hypothalamus (Hc) (the images were constructed from the selected z-stacks). Black dashes in the graphical illustrations outline the regions of interest. The white dashes outline the regions of higher magnification that were constructed from the selected z-stacks. Abbreviations: D: Dorsal telencephalic area; Dc: Central zone of dorsal telencephalic area; DI: later zone of dorsal telencephalic area; Dm: Medial zone of dorsal telencephalic area; Dp: Posterior zone of dorsal telencephalic area; LR: Lateral recess of diencephalic ventricle; LOT: Lateral olfactory tract; MOT: Medial olfactory tract; PR: posterior recess of diencephalic ventricle; Vc: Central nucleus of ventral telencephalic area; Vd: Dorsal nucleus of ventral telencephalic area; VI: Lateral nucleus of ventral telencephalic area; Vv: Ventral nucleus of ventral telencephalic area; DIV: Diencephalic ventricle; Hc: Caudal zone of periventricular hypothalamus; Hd: Dorsal zone of periventricular hypothalamus; PGZ: Periventricular gray zone of optic tectum; PTN: Posterior tuberal nucleus; TeO: Tectum opticum; TeV: Tectal ventricle; TL: Torus longitudinalis; TPp: Periventricular nucleus of posterior tuberculum.

Next, we aimed to identify the cell types that were expressing *gdnf* by performing FISH in combination with immunohistochemistry. Firstly, we investigated whether radial glial cells expressed *gdnf*. Here, we used glial fibrillary acidic protein (GFAP) and brain lipid-binding protein (BLBP) antibodies as markers for radial glial cells. As shown in Figure 2A–G, radial glial cells reside along the ventricular cavities in the telencephalon and co-expressed *gdnf*. However, there were only a few BLBP+/*gdnf*+ cells observed in the lateral margin of the periventricular gray zone of the optic tectum (PGZ), which is recognized as having quiescent radial glial cells (Figure 2H–N). In addition, BLBP+ cells in the caudal zone of the periventricular hypothalamus (Hc), a region of active proliferation, and neurogenesis were found to express *gdnf* transcripts (Figure 2P–S). 

**Figure 3:**
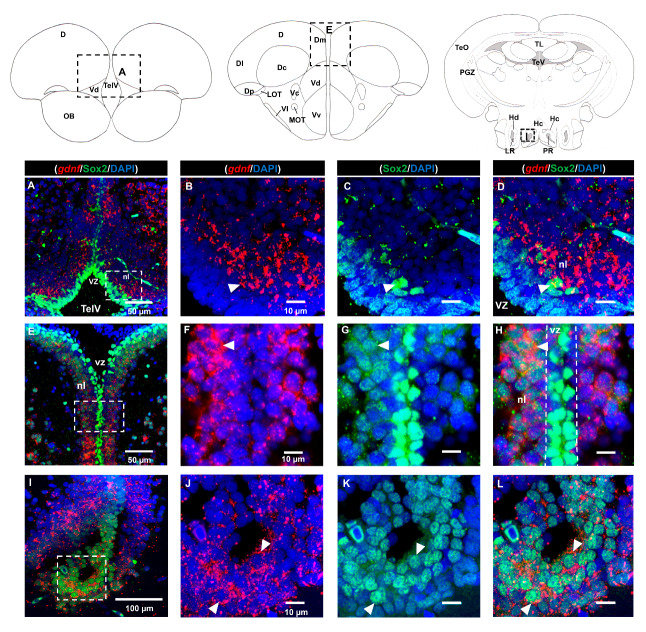
Co-localization of *gdnf* with Sox2. *gdnf* transcripts are found in Sox2-positive cells in the neuronal layer (nl) of (**A**–**D**) the dorsal nucleus of the ventral telencephalic area (Vd), (**E**–**H**) nl along the ventricular zone (vz)of the telencephalon and (**I**–**L**) the caudal zone of the periventricular hypothalamus (Hc). Black dashes in the graphical illustrations outline the regions of interest. White dashes outline the respective magnified regions that were constructed from the selected z-stacks. The white arrowheads indicate co-expression of *gdnf* with Sox2 immunoreactive cells. Abbreviations: D: Dorsal telencephalic area; Dc: Central zone of dorsal telencephalic area; DI: later zone of dorsal telencephalic area; Dm: Medial zone of dorsal telencephalic area; Dp: Posterior zone of dorsal telencephalic area; Hc: Caudal zone of periventricular hypothalamus; Hd: Dorsal zone of periventricular hypothalamus; LOT: Lateral olfactory tract; LR: Lateral recess of diencephalic ventricle; MOT: Medial olfactory tract; nl: neuronal layer; OB: Olfactory bulb; PGZ: Periventricular gray zone of optic tectum; PR: posterior recess of diencephalic ventricle; TelV: Telencephalic ventricle; TeO: Tectum opticum; TeV: Tectal ventricle; TL: Torus longitudinalis; Vc: Central nucleus of ventral telencephalic area; Vd: Dorsal nucleus of ventral telencephalic area; VI: Lateral nucleus of ventral telencephalic area; Vv: Ventral nucleus of ventral telencephalic area; vz: ventricular zone.

As we observed strong expression of *gdnf* in the ventricular region and its co-expression with BLBP in the telencephalon where stem cells are positioned, we attempted to ascertain whether the *gdnf*-expressing cells could be Sox2-positive stem cells. Strong Sox2 signals were observed in the ventricular zone (vz), ventral nucleus (Vv), dorsal nucleus (Vd) of the ventral telencephalon, and medial zone of dorsal telencephalon (Dm), with relatively weaker Sox2 signals in the neuronal layer (nl) (Figure 3A–H). Expression of *gdnf* was not detected in Sox2-expressing cells in the vz within the rostral subpallium (Figure 3A–D). Interestingly, *gdnf* expression was not co-localized with that of Sox2 in the vz but was seen in the neuronal layer (nl) in the telencephalon (Figure 3E–H). In the Hc, where *gdnf*+/BLBP+ cells reside, *gdnf* was also present in Sox2 immunoreactive cells (Figure 3I–L).

**Figure 4:**
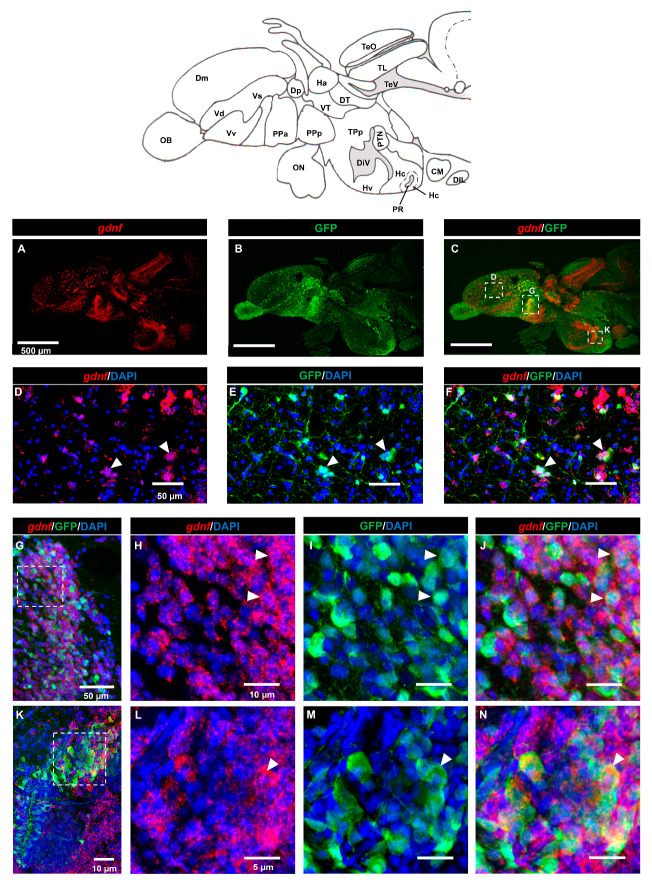
Expression of *gdnf* in GABAergic precursor cells of Tg (*dlx6a-1.4kbdlx5a/6a:* green fluorescence protein (GFP) fish. (**A**–**C**) An overview of *gdnf* transcripts (red) and GFP on a sagittal view of the adult zebrafish brain. The images depict co-localization of *gdnf* with subpopulation of GFP-positive cells in (**D**–**F**) pallium (**G**–**J**) parvocellular preoptic nucleus, anterior part (PPa), and (**K**–**N**) caudal zone of periventricular hypothalamus (Hc) as indicated with white arrowhead. The white dashes outline the enlarged regions that were constructed from selected z-stacks. Abbreviations: CM: Corpus mamillare; Dm: medial zone of dorsal telencephalic area; DIL: Diffuse nucleus of the inferior lobe; Dp: Posterior zone of dorsal telencephalic area; DiV: Diencephalic ventricle; Ha: Habenula; DT: Dorsal thalamus; Hc: Caudal zone of periventricular hypothalamus; Hv: Ventral zone of periventricular hypothalamus OB: Olfactory bulb; ON: Optic nerve; PPa: Parvocellular preoptic nucleus, anterior part; PPp: Parvocellular preoptic nucleus, posterior part; PR: Posterior recess of diencephalic ventricle; PTN: Posterior tuberal nucleus; TeO: Tectum opticum; TeV: Tectal ventricle; TPp: Periventricular nucleus of posterior tuberculum; V: ventral telencephalic area; Vd: dorsal nucleus of V; Vv: Ventral nucleus of V; Vs: Supracommissural nucleus of V; VT: Ventral thalamus.

GDNF was shown to be produced by GABAergic neurons in the rodent striatum [28], but it is not known if GDNF is expressed in GABAergic progenitor cells. Here, we used adult transgenic Tg (*dlx6a-1.4kbdlx5a/6a:*GFP) zebrafish for immunostaining with GFP and FISH for *gdnf* transcripts. Analysis of sagittal sections (Figure 4A–C) demonstrated that co-expression of *gdnf* with GFP was detected in the dorsal telencephalic area (Figure 4D–F), in the anterior part of the parvocellular preoptic nucleus (PPa) (Figure 4G–J), and in the Hc (Figure 4K–N).

### 3.3. gdnf is Synthesized in Both Early Differentiated Neurons and Mature Neurons 

**Figure 5:**
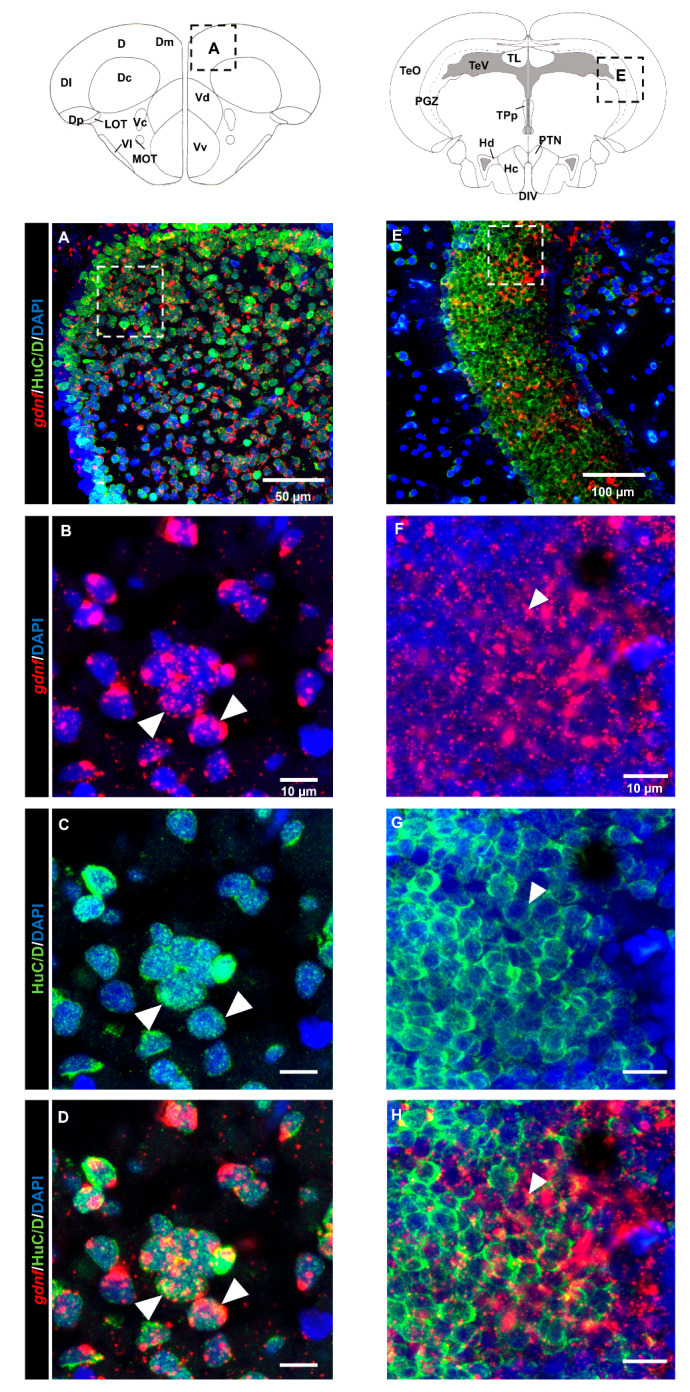
Coronal sections of adult brain revealing early-differentiated neuronal marker (HuC/D) immunoreactivity co-localized with *gdnf* mRNA. *gdnf* mRNA is co-expressed with HuC/D as indicated with white arrowheads in (**A**–**D**) dorsal telencephalic area, and (**E**–**H**) periventricular gray zone (PGZ) of the optic tectum. Black dashes in the graphical illustrations outline the regions of interest. The white dashes outline the enlarged regions that were constructed from selected z-stacks. Abbreviations: D: Dorsal telencephalic area; Dc: Central zone of dorsal telencephalic area; DI: later zone of dorsal telencephalic area; Dm: Medial zone of dorsal telencephalic area; Dp: Posterior zone of dorsal telencephalic area; LOT: Lateral olfactory tract; MOT: Medial olfactory tract ; Vc: Central nucleus of ventral telencephalic area; Vd: Dorsal nucleus of ventral telencephalic area; VI: Lateral nucleus of ventral telencephalic area; Vv: Ventral nucleus of ventral telencephalic area; DIV: Diencephalic ventricle; Hc: Caudal zone of periventricular hypothalamus; Hd: Dorsal zone of periventricular hypothalamus; PGZ: Periventricular gray zone of optic tectum; PTN: Posterior tuberal nucleus; TeO: Tectum opticum; TeV: Tectal ventricle; TL: Torus longitudinalis; TPp: Periventricular nucleus of posterior tuberculum.

**Figure 6:**
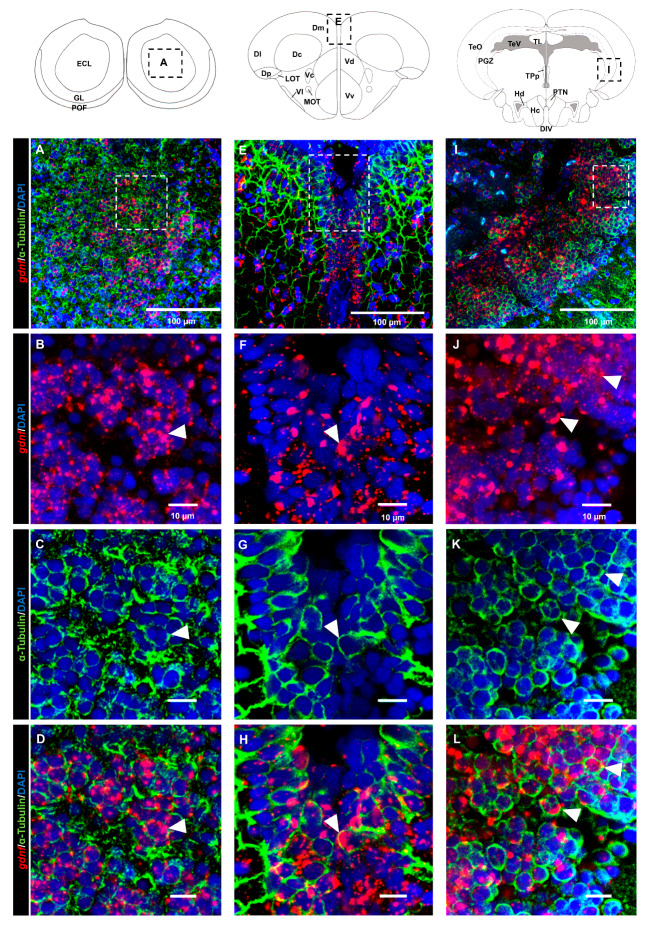
The mature neuronal marker acetylated α-tubulin is co-expressed with *gdnf* transcripts. Co-expression of *gdnf* and acetylated tubulin is detected in (**A**–**D**) olfactory bulb (OB), (**E**–**H**) medial zone of the dorsal telencephalon area (Dm), and (**I**–**L**) periventricular gray zone (PGZ) of optic tectum. Black dashes in the graphical illustrations outline the regions of interest. White dashes outline the respective magnified regions that were constructed from selected z-stacks. The white arrowheads indicate the co-expression of *gdnf* and acetylated α-tubulin. Abbreviations: ECL: External cellular layer of olfactory bulb including mitral cells; GL: Glomerular layer of olfactory bulb; POF: Primary olfactory fiber layer; D: Dorsal telencephalic area; Dc: Central zone of dorsal telencephalic area; DI: later zone of dorsal telencephalic area; Dm: Medial zone of dorsal telencephalic area; Dp: Posterior zone of dorsal telencephalic area; LOT: Lateral olfactory tract; MOT: Medial olfactory tract; Vc: Central nucleus of ventral telencephalic area; Vd: Dorsal nucleus of ventral telencephalic area; VI: Lateral nucleus of ventral telencephalic area; Vv: Ventral nucleus of ventral telencephalic area; DIV: Diencephalic ventricle; Hc: Caudal zone of periventricular hypothalamus; Hd: Dorsal zone of periventricular hypothalamus; PGZ: Periventricular gray zone of optic tectum; PTN: Posterior tuberal nucleus; TeO: Tectum opticum; TeV: Tectal ventricle; TL: Torus longitudinalis; TPp: Periventricular nucleus of posterior tuberculum.

Analysis of the brain regions in the dorsal telencephalon (Figure 5A–D) and PGZ (Figure 5E–H) that labeled with the HuC/D marker suggested that most of the *gdnf*-positive cells were committed toward neuronal linage. This evidence was confirmed by co-labeling of *gdnf* mRNA in mature neuronal cells with anti-acetylated α-tubulin antibody in the OB (Figure 6A–D), dorsal telencephalon (Figure 6E–H), and PGZ (Figure 6I–L). 

### 3.4. gdnf is Co-Expressed in the Subpopulation of Tyrosine Hydroxylase Immunoreactive Neurons

**Figure 7:**
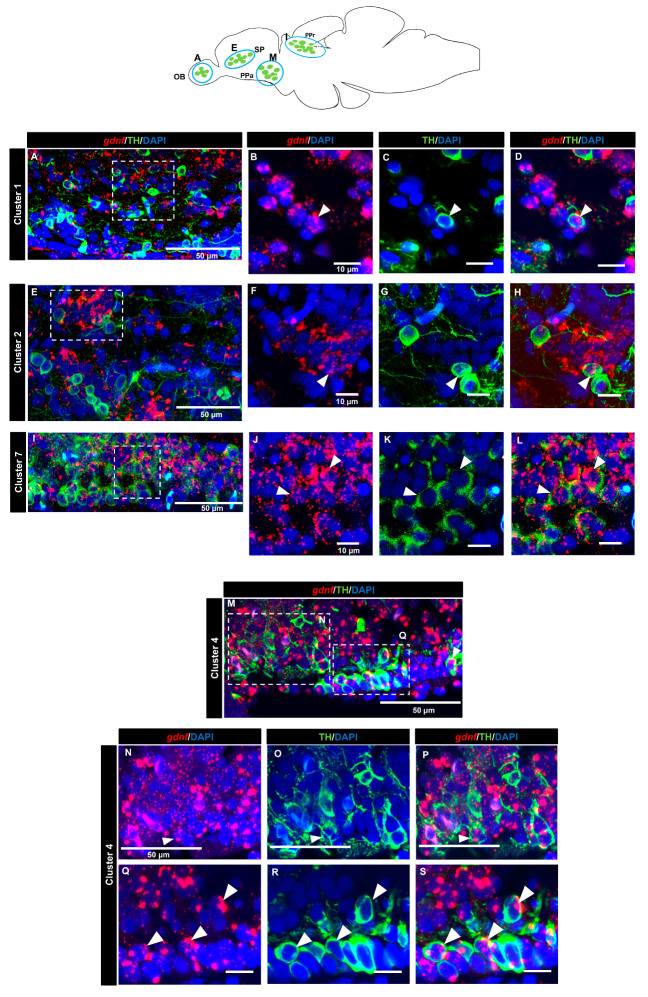
*gdnf* is expressed in catecholaminergic subpopulations in the telencephalon and pretectum region. Sagittal section of adult zebrafish brain showing some co-expression of *gdnf* in TH-positive neurons of (**A**–**D**) cluster 1 in olfactory bulbs (OB), and (**E**–**H**) cluster 2 in subpallium (SP). Clear co-expression is observed in (**I**–**L**) cluster 7 in periventricular pretectal nucleus (PPr) and (**M**–**S**) cluster 4 in parvocellular preoptic nucleus, anterior part (PPa). The white arrowheads represent the co-expression of *gdnf* and TH. Blue outlines in the graphical illustration represent the regions of the respective TH subpopulation neurons. White dashes outline the respective magnified regions that were constructed from selected z-stacks.

**Figure 8:**
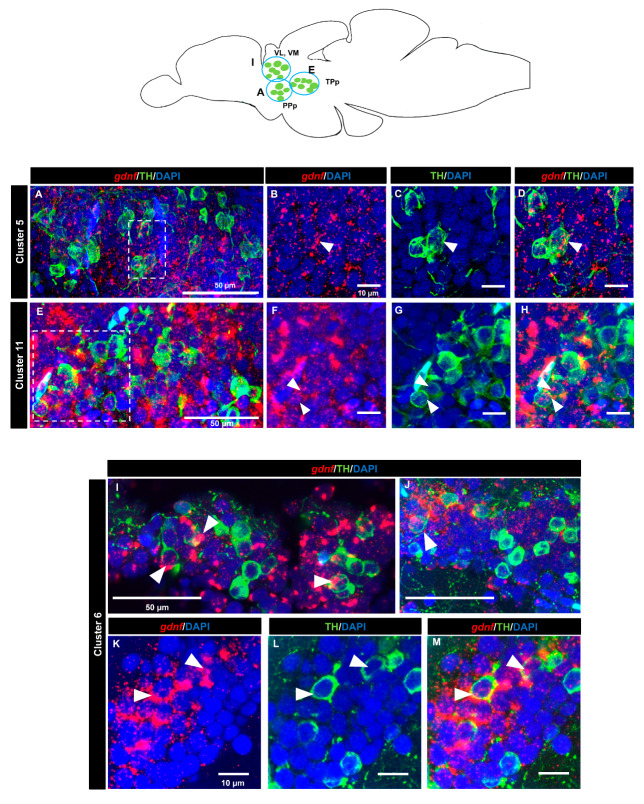
Co-expression of *gdnf* mRNA with catecholaminergic subpopulations in the diencephalon. FISH/immunohistochemistry for *gdnf* (red) and TH (green) on sagittal sections of (**A**–**D**) cluster 5 in parvocellular preoptic nucleus, posterior part (PPp), (**E**–**H**) cluster 11 in periventricular nucleus of posterior tuberculum (TPp), and (**I**–**M**) cluster 6 in anterior, intermediate, ventrolateral, and ventromedial thalami nuclei (A, I, VL, and VM). Blue circles in the schematic sagittal view of the adult zebrafish brain represent the regions of the respective TH neuron subpopulations. White dashes outline the respective magnified regions that are constructed from selected z-stacks. The white arrowheads indicate the co-localization of *gdnf* and TH.

**Figure 9:**
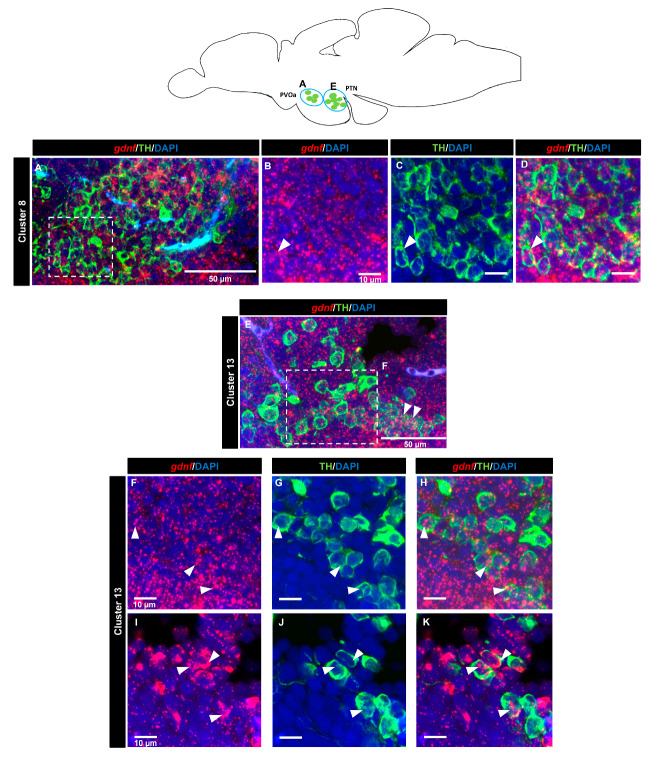
*gdnf* mRNA is detected in catecholaminergic subpopulations in the ventral diencephalon. Distribution of neuronal clusters expressing *gdnf* (red) and TH (green) of (**A**–**D**) cluster 8 in the paraventricular organ, anterior part (PVOa) and (**E**–**K**) cluster 13 in the periventricular hypothalamus and posterior tuberal nucleus (PTN). Blue circles in the schematic sagittal view of the adult zebrafish brain represent the regions of the respective TH subpopulation neurons. White dashes outline the magnified regions that were constructed from selected z-stacks. The white arrowheads indicate the co-expression of *gdnf* and TH.

A tyrosine hydroxylase (TH) antibody was used as a marker for dopaminergic neurons. In the OB (cluster 1) (Figure 7A–D) and the subpallium area of the telencephalon (cluster 2) (Figure 7E–H), a few cells expressing *gdnf* transcripts also expressed TH. In the diencephalon, we did not see co-localization in cluster 3 (preoptic area). Co-expression of *gdnf* with TH-positive neurons was observed in cluster 7 of the periventricular pretectal nucleus (PPr) (Figure 7I–L) and in cluster 4 in the anterior part of the parvocellular preoptic nucleus (PPa) (Figure 7M–S). Only a few cells of cluster 5 in the posterior part of parvocellular preoptic nucleus (PPp) co-expressed *gdnf* and TH (Figure 8A–D), whereas a more occasional co-expression was seen in cluster 11 (Figure 8E–H), cluster 6 (Figure 8I–M) in the prethalamic region, cluster 8 in the anterior part of the paraventricular organ (PVOa) (Figure 9A–D), and cluster 13 of the posterior tuberal nucleus (PTN) (Figure 9E–K). Transcripts of *gdnf* were not detected in clusters 9 and 12. Table 2 summarizes the results. 

## 4. Discussion

The present study describes the expression pattern of *gdnf* transcripts at the cellular level in the adult zebrafish brain. In the absence of a specific antibody to zebrafish gdnf, we used in situ hybridization in combination with immunohistostaining to further identify sub-population of neurons that express *gdnf*. Our results demonstrated that *gdnf* transcripts were not only localized in progenitor/radial glial cells but also in neurons, such as dopaminergic neurons. 

Our results concur with a previous study that demonstrated that *gdnf* expression persists into adulthood and is not restricted to developmental stages [29]. Likewise, GDNF expression has been reported in rodents at adult stages [30,31,32]. The wide expression of *gdnf* in the zebrafish brain suggests a role for *gdnf* in maintaining neuron survival and in other physiological functions in the central nervous system. 

Heterogenous stem/progenitor cells reside in the ventricular zone of the telencephalon and are known to be responsible for adult neurogenesis and regeneration in zebrafish [33,34]. Our in situ hybridization results revealed there was intense *gdnf* expression along the periventricular zone, leading us to look into the localization of *gdnf* in neuronal progenitor cells (NPCs) and radial glial cells.

Sox2 is present in stem cells/progenitor cells of the central nervous system (CNS) and is required for maintaining neural stemness (pluripotency and self-renewal capacities) and mending neural injuries [35,36,37]. It is interesting to note that *gdnf* is only expressed in the Sox2 immunoreactive cells located in the neuronal layer, adjacent to the periventricular zone of the telencephalon. Prior to neuronal differentiation, Sox2 expression is suppressed in order to activate the proneural transcription program. Our results showed that cells that are *gdnf-*positive express an apparently much lower Sox2 signal compared to that seen in the ventricular zone (Figure 3E–H). This would indicate that these cells were early committed neurons. GDNF is a protein that is mainly secreted by neurons in the mammalian CNS [30,38]. We have similarly observed in this study that *gdnf* expression was mostly localized with early differentiated neurons and mature neurons, further supporting the notion that *gdnf* enhances neuronal differentiation [39,40,41]. It could also be involved in early neuronal fate specification during adult brain neurogenesis. 

It is unclear whether rodents express GDNF in radial glial cells despite some studies suggesting this possibility [12,42,43,44]. Some groups showed that there was low expression of GDNF by glial cells [28,30,45]. In the zebrafish brain, we observed *gdnf* co-localized with radial glial cells (in GFAP and BLBP positive cells) in the ventricular telencephalic area (Vd/Vv) (Figure 2A–G). The zebrafish telencephalon, particularly along the ventricular zone, is a well-studied neurogenic niche. The telencephalon has tremendous neuro-regenerative capacity that could functionally help its recovery from lesions, with the ventricular radial glial cell being a key player in the process [46,47]. Increases in GDNF have been reported in synthesizing glial cells following brain lesions or in neuroinflammation animal models [38,48]. Based on these observations, it could be alluded that neuronal progenitor niche cells require *gdnf* for homeostatic maintenance in the telencephalon. In our study, it was unclear whether *gdnf*+/BLBP+ cells were state I (quiescent) or state II (dividing) radial glial cells, as state II cells are intermingled throughout the ventricular zone and it has been reported that less than 20% of the radial glial cells are expressing the proliferating cell nuclear antigen (PCNA) protein [49,50]. Notch activity and its receptors are present in quiescent radial glial cells and notch signaling appears to be highly associated with regulating the equilibrium between proliferative and quiescence stages of radial glial cells [49,50]. A recent study in rats showed that GDNF could counteract spinal cord injury-induced Notch activation and promote transplanted neuronal progenitor cell differentiation toward neuronal linage rather than into astrocytes [51]. A mechanistic study on the relationship of *gdnf* and Notch signaling in radial glial cells fate in the zebrafish brain would be of interest. Likewise, the radial glial cells in the periventricular gray zone (PGZ) are characterized as quiescent and injury-inducible to become active proliferative cells [52,53,54]. However, in our hands, we detected few BLBL+/*gdnf*+ cells occasionally in the lateral margin of the PGZ. In the hypothalamus, particularly in the caudal zone of the periventricular hypothalamus (Hc), *gdnf* is co-localized with BLBP immunoreactive cells and Sox2. Diversity of *gdnf* expression pattern in the brain suggested it has multiple roles in maintaining radial glial cells.

The Dlx family of homeobox transcription factors are expressed in progenitor cells that give rise to GABAergic neurons in vertebrates, including zebrafish [55,56,57]. Expression of *dlx5a* partially overlaps with that of *gad1*, a GABAergic neurons marker. Hence, the co-localization of *gdnf* in *dlx5a*-positive neurons of Tg (dlx5a/dlx6a:EGFP) transgenic fish supports our hypothesis that *gdnf* is present in GABAergic precursor cells. This is also consistent with the evidence in rodent that GABAergic interneurons are a major source of GDNF [28,58].

Bromodeoxyuridine (BrdU) pulse-chase assays revealed that ventricular progenitor cells not only give rise to GABAergic interneurons but also to tyrosine hydroxylase-positive neurons that migrate a short distance into the parenchyma (neuronal zone) [59]. It is known that GDNF is expressed in tyrosine hydroxylase-positive neurons in rodents [30,60] as well as in the human substantia nigra [61]. We wanted to investigate if a similar phenomenon occurs in the zebrafish dopaminergic system. Groups of dopaminergic neurons have been identified and are distributed rostral to caudal in the zebrafish brain [62,63,64,65,66]. We provide the first evidence that *gdnf* is co-expressed with TH-immunoreactive neurons in subsets dopaminergic neuron clusters, including group 13 in the ventral diencephalon, similar to what was previously documented in the adult rat brain [67]. Loss of dopaminergic neurons in ventral diencephalon was shown to result in locomotor defects after neurotoxin insult [68,69,70,71,72,73] or after genetic disruption [74,75,76,77]. 

Homology between zebrafish and mammals prompted the question of whether *gdnf* was involved in dopaminergic neuron function. GDNF is an important molecule for *in vitro* induction of dopaminergic neuron differentiation [78,79], and overexpression of GDNF in stem cell-based therapy improved dopaminergic neuron commitment after transplantation [41,80,81]. Although exogenously applied GDNF was shown to be neuroprotective or restorative in PD models [58], the endogenous function of GDNF in dopaminergic neurons development and survival remains controversial [11,16]. Nonetheless, a recent study using a novel transgenic model approach provided evidence of GDNF as indispensable for catecholaminergic neuron maintenance [9]. Our laboratory had also explored *gdnf* function in zebrafish crispants. Impaired *gdnf* function in zebrafish crispants led to a decrease in dopaminergic neurons in clusters 8 and 13 during development [73]. Whether there would be any spatial or temporal differences in *gdnf* expression patterns in dopaminergic progenitor cells after damage would be an interesting question to address and could provide clues on *gdnf* regulation of dopaminergic neuron regeneration in zebrafish. 

## 5. Conclusions

This study provided insight into the anatomical distribution of GDNF in the adult zebrafish brain, wherein the *gdnf* gene was mainly expressed in early committing and mature neurons, partly in radial glial cells, but not in Sox2-positive neuronal stem cells in the ventral zone of the telencephalon. Our results may shed some light on the potential role of GDNF in adult neurogenesis and regeneration. Importantly, we demonstrated a link between *gdnf* expression and GABAergic precursor cells and dopaminergic neurons. 

## Figures and Tables

**Table 1:** Antibodies utilized for immunohistochemistry.

Host	Target	Dilution	Catalog
Mouse	HuC/HuD	1:200	A-2127 (Invitrogen)
Rabbit	Sox2	1:200	Ab97959 (Abcam)
Rabbit	Tyrosine hydroxylase (TH)	1:200	AB152 (Merck Millipore)
Mouse	Green fluorescence protein (GFP)	1:200	632381 (Clontech)
Rabbit	Brain lipid-binding protein (BLBP)	1:200	ABN14 (Merck Millipore)
Rabbit	Glial fibrillary acidic protein (GFAP)	1:200	Z0334 (Dako)
Mouse	Acetylated α-tubulins	1:200	T7451 (Sigma-Aldrich)

**Table 2:** Qualitative analysis of *gdnf* expression in TH immunoreactive neurons in the adult zebrafish brain.

	Brain Areas	TH+ Groups	Expression Level
Telencephalon	Olfactory bulb (OB)	Cluster 1	+
	Subpallium (SP)	Cluster 2	+
Diencephalon	Preoptic area	Cluster 3	ND
		Cluster 4	+++
	Prethalamic	Cluster 5	+
		Cluster 6	++
		Cluster 11	++
	Periventricular pretectal nucleus (PPr)	Cluster 7	+++
	Anterior part of paraventricular organ (PVOa)	Cluster 8	++
	Intermediate part of paraventricular organ (PVOi)	Cluster 9	ND
	posterior part of paraventricular organ (PVOp)	Cluster 10	+
	Posterior tuberculum (TP)	Cluster 12	ND
	Posterior tuberal nucleus (PTN)	Cluster 13	++

+: Few cells; ++: moderate number of cells; +++: numerous cells; ND: not detected/low expression.
